# Age-Related Changes as the Primary Driver of Pineal Gland Involution—A Morphological Study in Health and Disease

**DOI:** 10.3390/ijms27073093

**Published:** 2026-03-28

**Authors:** Olga Junemann, Dmitry Otlyga, Inna Bukreeva, Sergey Saveliev

**Affiliations:** 1 Institute of Nanotechnology-CNR (Consiglio Nazionale delle Ricerche), Piazzale Aldo Moro 5, 00185 Rome, Italy; inna.bukreeva@cnr.it; 2 Avtsyn Research Institute of Human Morphology of Federal State Budgetary Scientific Institution “Petrovsky National Research Centre of Surgery”, 117418 Moscow, Russia; otlyga@bk.ru (D.O.); embrains@mail.ru (S.S.)

**Keywords:** human pineal gland, astroglia, glial cyst, normal aging, neurodegenerative diseases, mental disorders

## Abstract

The human pineal gland (PG) undergoes structural and cellular changes during the aging process, yet the underlying patterns and mechanisms remain insufficiently understood. In this study, we analyzed the lobular architecture and astrocytic network of the PG and identified two distinct pathways associated with normal aging. The first is characterized by an increase in astrocyte number within the pineal parenchyma, suggesting a compensatory role in supporting pinealocyte function. The second pathway involves disruption of the lobular structure, leading to a decline in the functional integrity of the gland. While pathological conditions such as neurodegenerative and psychiatric disorders may accelerate pineal degeneration and reduce melatonin production, our results suggest that normal aging is the principal factor driving this involutional process. These findings contribute to a deeper understanding of the morphological aging pathways of the pineal gland and their potential functional implications.

## 1. Introduction

The pineal gland (PG) is an endocrine organ in the brain, primarily composed of pinealocytes (about 95% of the cells); the rest are mainly astrocytes and microglia embedded in a network of blood vessels and nerve fibers [1]. Pinealocytes produce melatonin, which plays an important role in the human body. In addition to day-and-night rhythm maintenance, melatonin acts as an antioxidant [2,3,4], slows down tumor development [5,6], participates in thermoregulation [7] and reproduction [8,9,10] and can serve as a protection against fibrosis [11].

Astrocytes have numerous functions in the central nervous system. Astrocytes maintain the molecular homeostasis of the central nervous system (CNS) by transporting important ions and protons and degrading and catabolizing neurotransmitters [12,13]. They determine the cytoarchitecture of the gray matter by lining it and establishing contacts with the vascular system and controlling the blood–brain barrier (BBB), which, together with microglia, represents the most important defense system of the CNS [12]. Some authors claim that the PG has a similar structural organization and relationship between the pinealocytes and astrocytes as neurons and astrocytes [14,15,16]. According to some data, astrocytes can also synthesize melatonin [6,17]. In addition, it is assumed that astrocytes in PG can also take up and transport serotonin [16], thus participating in neurotransmission.

Numerous studies state changes in the human PG as a result of aging and some neurodegenerative and mental pathologies. Impairment in melatonin synthesis and/or subsequent free radical damage due to decreasing activity of melatonin as an antioxidant has been reported by R. Reiter [2]. Such changes are often associated with calcification [18,19,20,21,22]. Alterations in the PG have also been studied in Alzheimer’s disease [23,24,25,26] and schizophrenia [27,28,29]. As mentioned above, most studies focus on concrement formation. A relatively understudied issue is the alteration of the lobular structural organization of the human PG and its potential impact on glandular function. In our previous study, we have alluded to this process [30]. To date, however, a potential relationship between the astrocytic network and the lobular organization of the gland remains insufficiently explored. Our aim is to study the astrocyte network of the human PG in normal aging and in the contexts of neurodegenerative (vascular dementia, Alzheimer’s disease) and mental (schizophrenia, alcoholism) diseases, with a focus on its relation to changes in the pineal parenchymal organization.

## 2. Results

### 2.1. Age-Related Variability

**Figure 1:**
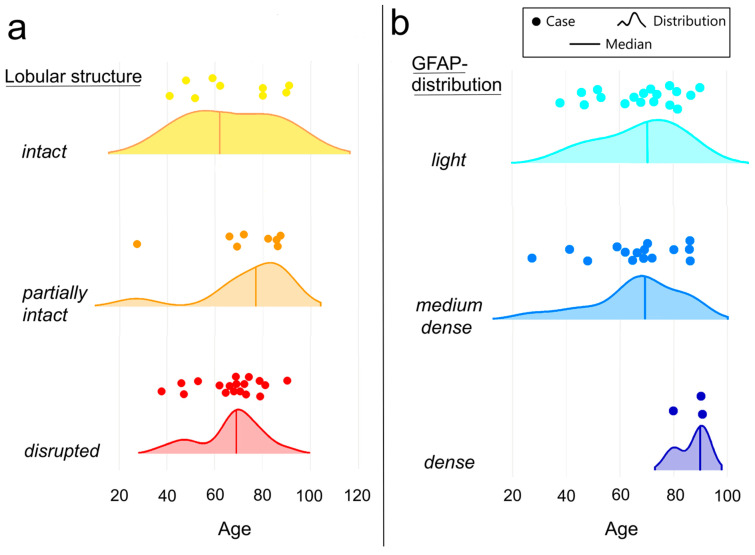
Control group. (**a**) Age-related variability in lobular organization—all three types of lobular organization can be found across the examined age range, the lobular organization types *partially intact* and *disrupted* occur more frequently in older people; (**b**) age-related variability in GFAP distribution—both variants *(light* and *medium dense*) occur across the examined age range with weak tendency to occur more frequently in older people for *medium dense* variant. Astrocyte network type *dense* was only found in 3 cases among older people.

To investigate age-related variability in the astrocyte network and lobular organization, we analyzed PGs from 36 individuals aged 27 to 91 years without mental or neurodegenerative disorders. We detected the lobular organization variant *intact* and *partially intact* in nine cases for each of them and the *disrupted* variant in 18 cases. Figure 1a shows that all three types of lobular organization can be found across the examined age range. Nevertheless, there are differences in the shape of the distribution—the density curve is skewed to the right for the lobular organization types *partially intact* and *disrupted*, which means that these two variants occur more frequently in older people.

Figure 1b shows the relationship between the density of the astrocyte network and age. In 18 cases, the astrocyte network density can be defined as *light* and in 15 cases as *medium dense*. Both variants occur across the examined age range, the density curve is only slightly skewed to the right in the *medium dense* variant (i.e., shows the weak tendency to occur more frequently in older people). It is remarkable that the astrocyte network type *dense* was only found in 3 cases among older people (80 years and older).

**Figure 2:**
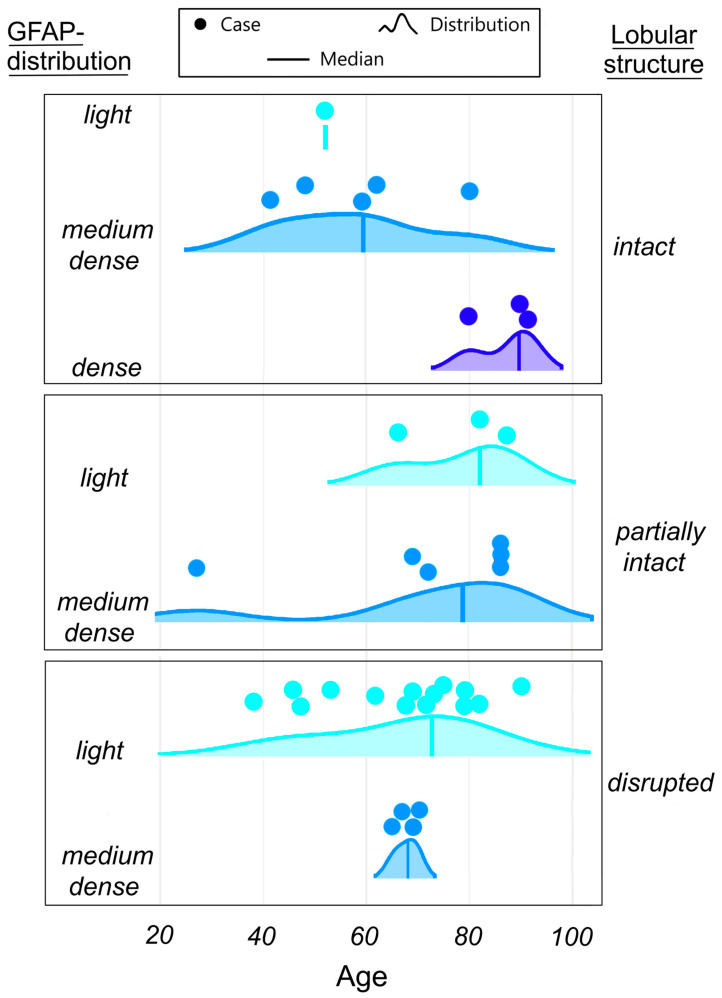
Age-related changes in coincidence between lobular organization and astrocyte network density in control group—only one case of *light* GFAP distribution detected in the *intact* lobules, type *medium dense* occurs more frequently and seems to be independent of age, variant *dense* found only in older persons. The *light* type of lobular organization appears in older people, the density curve for the *partially intact* lobular type shows a strong tendency to appear in older patients, the *medium dense* type of lobular organization is detected only in older persons.

As a next step, we analyzed the coincidence between lobular organization and astrocyte network density. The results are shown in Figure 2. Only one case of light GFAP distribution was detected in the intact lobules. The distribution type medium dense occurs more frequently (five cases) and seems to be independent of age. Variant dense (three cases) was only found in older persons (80 years and older). For the variant of lobular organization partially intact, three cases were defined as light and six cases as a medium dense GFAP distribution type. The light type of lobular organization appears in older people (over 60 years old). The density curve for the partially intact lobular type is skewed to the right, thus showing a strong tendency to appear in older patients. In the variant of lobular organization damage, 14 cases of light GFAP distribution type were found. This type of distribution can be observed in all age groups. The medium dense type of lobular organization is detected in four cases and only in older persons (over 60 years old).

Thus, it is worth noting that the astrocyte network variant *dense* is only detected in *intact* lobules. The *light* astrocyte network is found most frequently in *disrupted* lobules in all age groups with a weak tendency to appear in older age. The *light* astrocyte network is detected in the *intact* lobules only in one case.

### 2.2. Pathological Variability

**Figure 3:**
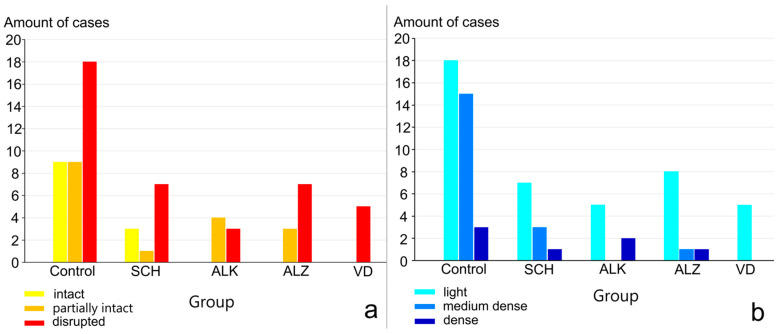
Pathological variability: (**a**) lobular structure organization—variant *disrupted* is the most common in each group analyzed. Type *intact* found only in control und schizophrenia groups, in dementia group found only *disrupted* variant; (**b**) organization of the astrocyte network—*light* structure is the most frequently observed in all groups, the dementia group shows only *light* form of astrocytic organization.

We then analyzed the pathological variability of both parameters and compared them with values of the control group. From Figure 3a, it can be seen that the lobular organization variant *disrupted* is the most common in each group analyzed. It is remarkable that in groups alcoholism, vascular dementia and Alzheimer’s disease, the lobular organization type *intact* could not be detected at all, whereas in the dementia group only the *disrupted* variant was found.

As shown by the organization of the astrocytic network (Figure 3b), the *light* structure is the most frequently observed in all groups. Remarkably, the dementia group shows only one form of astrocytic organization, namely *light*.

Table 1 presents all results in case numbers and percentages.

**Figure 4:**
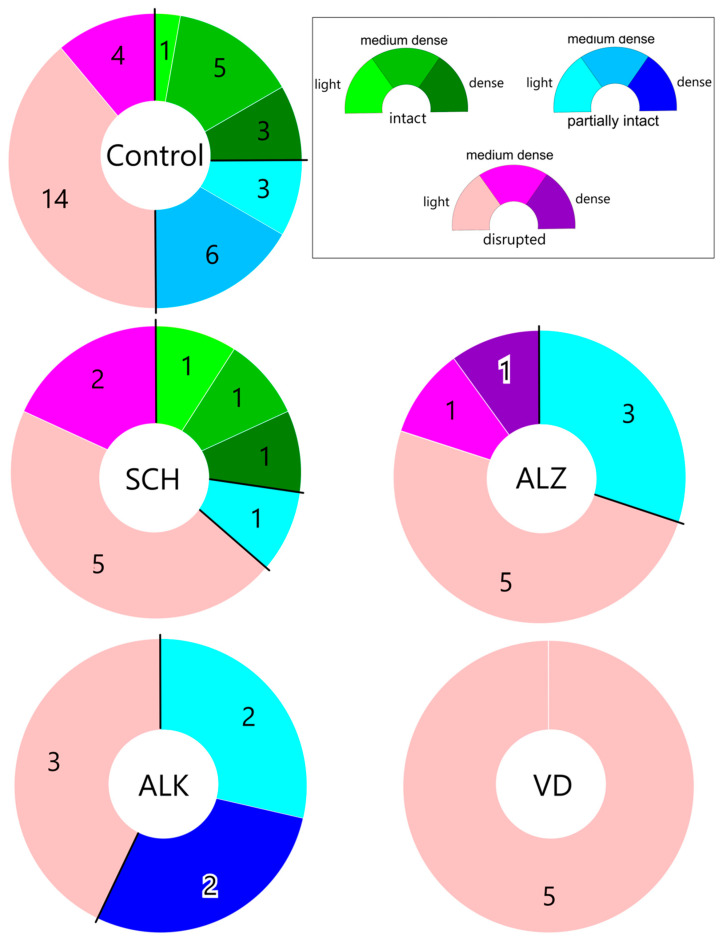
Lobular structure and astrocyte network density with the numbers of cases in each combination—coincidence between *disrupted* lobular organization variant and *light* astrocyte network is the strongest in all groups. Semicircles indicate the association of the GFAP distribution type (*light, medium dense* or *dense*) with various types of lobular structure (*intact, partially intact* or *disrupted*).

Similarly to the control group, we analyzed and compared the coincidence between lobular structure and astrocyte network density for all groups; the results are shown in Figure 4. Notably, the coincidence between the *disrupted* lobular organization variant and the *light* astrocyte network is the strongest in all groups.

Table 2 shows the results of the coincidence analysis.

### 2.3. Statistical Analysis

Statistical analysis reveals significant associations among all examined parameters—health status and both lobular structure and GFAP distribution. Cramér’s V coefficients were 0.29, 0.30, and 0.41, respectively, indicating a medium to moderately large effect.

### 2.4. Glial Cysts

Glial cysts were found in all groups excluding the schizophrenia group. Table 3 presents cyst findings in each group. The formation of cysts does not appear to be related to age. It is worth noting that cysts are most common in the combination *disrupted* lobular organization–*light* astrocyte network.

**Figure 5:**
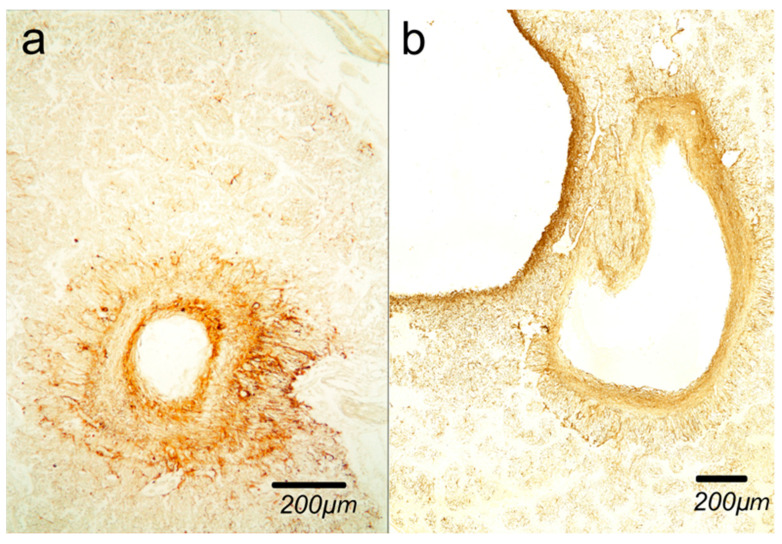
Glial cyst in pineal gland (**a**) by *light* astrocyte network; (**b**) by *medium dense* astrocyte network. Astrocyte network is clearly detectable in both cases. (Immunohistochemical staining with anti-GFAP antibody).

Figure 5 shows glial cysts by *light* and *medium dense* astrocyte network.

## 3. Discussion

### 3.1. Age-Related Variability

As we have indicated in our earlier work, the organization of the human pineal parenchyma shows a high degree of variability [30]. Although the slight accumulation of damaged lobules is found in older individuals, all three variants can be found at any age. We see a different picture when looking at the astrocyte network.

Although the *light* type is the most frequently observed, as illustrated in Figure 1b, the *dense* astrocytic network appears exclusively in older individuals. Additionally, the distribution of the *medium dense* variant shows a slight rightward shift, indicating a higher prevalence among the elderly. These findings may raise the possibility that astrocytes play a more prominent role as pinealocyte activity declines; however, this interpretation remains speculative and requires further functional investigation.

It should be noted that GFAP immunoreactivity does not directly reflect astrocyte number, but rather their structural organization and activation state. The considerable variability observed across samples, particularly in older individuals, suggests that pineal aging may not follow a uniform trajectory. Instead, different patterns of structural remodeling may occur, possibly reflecting distinct modes of aging in the human pineal gland.

Numerous published data suggest that astrocytes produce melatonin [6,17] and can transport neurotransmitters such as serotonin [16]. This may also explain our results. Given that a dense astrocytic network is more frequently observed in older individuals, these findings may indicate age-related changes in the relative roles of astrocytes and pinealocytes; however, any functional implications remain speculative and require further investigation. In some cases, we observed a similar picture as Gómez Esteban with co-authors by studying gliosis in cow PG. They suggest that pineal gliosis could be related to age, which may influence decreasing melatonin levels in the CSF [31]. These results are broadly consistent with our findings.

When analyzing the coincidence of astrocyte network density and lobules condition, it is remarkable that a *dense* organization of the astrocytes only occurs in *intact* lobular parenchyma.

In contrast, *light* organization is most frequently found in damaged lobules. The vascular organization of the human pineal gland appears to be closely associated with its connective tissue septa. Previous studies have demonstrated that blood vessels are predominantly located within these septa and enter the gland along these structures, forming the interlobular vascular network [32,33]. In addition, connective tissue septa have been described as key structural components that compartmentalize the gland and serve as conduits for blood vessels and nerve fibers [33]. Against this background, structural alterations or progressive disintegration of septa, as observed in the present study, may have important implications for the organization of the pineal microvasculature. In particular, the disruption of septal architecture could contribute to alterations in the spatial distribution and integrity of the capillary network, potentially affecting tissue perfusion. However, the extent to which these structural changes directly impact vascular function remains to be elucidated and warrants further investigation.

### 3.2. Pathological Variability

Analysis of the pathological variability of the lobular structure and astrocyte network shows that the distribution of GFAP values is similar to that of normal aging.

The combination of a *disrupted* lobular structure and a light astrocyte network is the most frequently observed case across all groups and resembles the pattern present in the control group. However, the situation is different when the lobular organization of the parenchyma is considered. Remarkably, the *intact* type of the parenchymal structure is found only in people with schizophrenia, apart from the control group.

Instead, we found only *disrupted* lobular structure in people with VD. Of course, this could also be due to the smaller number of samples. On the other hand, according to a number of authors, people with AD and dementia often suffer from sleep disorders [23,34,35,36].

Some researchers report a decrease in melatonin production by the PG in schizophrenic patients [37,38], as well as in patients with alcoholism [39,40]. Based on the current state of knowledge, we believe that pathological conditions can influence the degeneration of the pineal gland structure and the consequent decrease in melatonin production. However, it is mainly the normal aging process that is responsible for pineal involution. Alzheimer’s disease itself is an example of such conditions. The processes that eventually lead to the disease also occur in normal aging, but are not as severe, and although they also impair cognitive function, they do not lead to the disease [41]. However, further research with larger samples is needed.

Statistical analysis reveals significant associations among all examined parameters. Cramér’s V coefficients indicate a medium to moderately large effect. This also highlights the need to analyze a greater number of participants.

### 3.3. Glial Cysts

In post-mortem examinations, glial cysts are found in 25–41% of otherwise normal PGs [42]. PG cysts usually have no clinical effect and remain asymptomatic for years [43] but, depending on their size, they can significantly reduce the number of synthetically active pinealocytes and thus melatonin production. The formation of cysts may also be associated with astrocytes. Cyst formation is described as follows. First, a so-called glial spot develops as a form of age-related involution of the pineal parenchyma. Parenchymal atrophy leads to diffuse glial proliferation, which can be seen as a plaque with tissue replacement for the perished pineal cells. At the center of these plaques, insufficient nutrient supply leads to cyst formation due to occlusion of the capillaries providing blood supply [44]. According to our findings, this process is not age-dependent; however, it is predominantly observed in pineal glands that show the combined pattern of a *disrupted* lobular structure and a *light* astrocytic network. A more-or-less thick layer of astrocytes persists along the periphery of the cysts, even if less astrocytes are found in the remaining parenchyma. The underlying mechanism of cyst formation remains unclear.

## 4. Materials and Methods

### 4.1. Materials

We examined PG specimens from 69 individuals divided into five groups—seven individuals with alcoholism (range 40–67 years), 11 individuals with schizophrenia (range 25–92 years), 10 individuals with Alzheimer’s disease (range 67–83 years), five individuals with vascular dementia (range 66–79 years), and 36 individuals of the control group (range 27–91 years). The control group included individuals without neurodegenerative or mental disorders.

Autopsy material was obtained from the collection of the Federal State Scientific Institution Research Institute of Human Morphology (Moscow, Russian Federation).

### 4.2. Methods

#### 4.2.1. Histology

Pineal glands were fixed in 10% buffered formaldehyde or Carnoy fluid, dehydrated in 8 portions of isopropyl alcohol and embedded in paraffin blocks. The paraffin blocks were cut into 7 µm sections and stained with Mallory’s method for connective tissue or with halocyanine to analyze the condition of the pineal gland lobules.

#### 4.2.2. Immunohistochemistry

**Figure 6:**
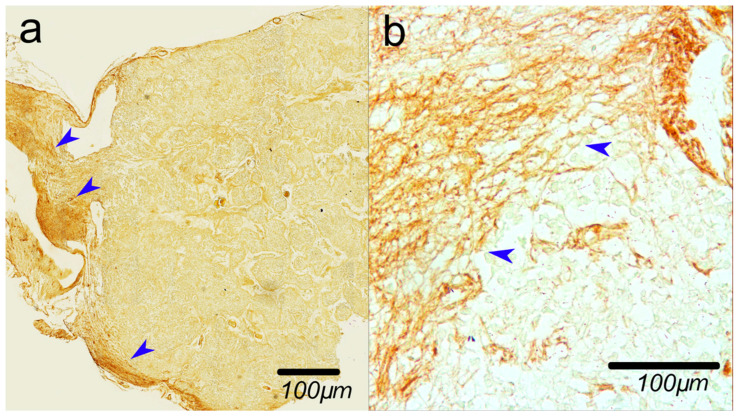
(**a**) Strong positive reaction in habenula accompanied by very weak reaction in pineal gland; (**b**) detailed view of GFAP-positive fibers. Blue arrows show the glial fibers. Immunohistochemical staining with anti-GFAP antibody.

To analyze the density of the astrocyte network, we used rabbit polyclonal antibodies to GFAP (Cat# PA5-16291, Invitrogen (Carlsbad, CA, USA), Dilution 1:200). As secondary antibodies, we used the UltraVision Quanto Detection System HRP DAB (Cat# TL-125-QHD, Thermo Fisher Scientific (Waltham, MA, USA)). For the analysis, we selected only the PGs paired with habenula (Figure 6) to perform positive control of GFAP antibodies for each sample, since the habenula contains numerous nerve fibers and a well-developed astrocyte network [45]. Staining for GFAP and staining for stromal component was performed on the adjusted sections on the same specimen of the pineal gland. The intensity of GFAP staining was evaluated using a semi-quantitative visual scoring system (0 = none/minimal, 1 = weak, 2 = moderate, 3 = strong), which is a commonly applied method in immunohistochemical studies, including for GFAP [46,47].

**Figure 7:**
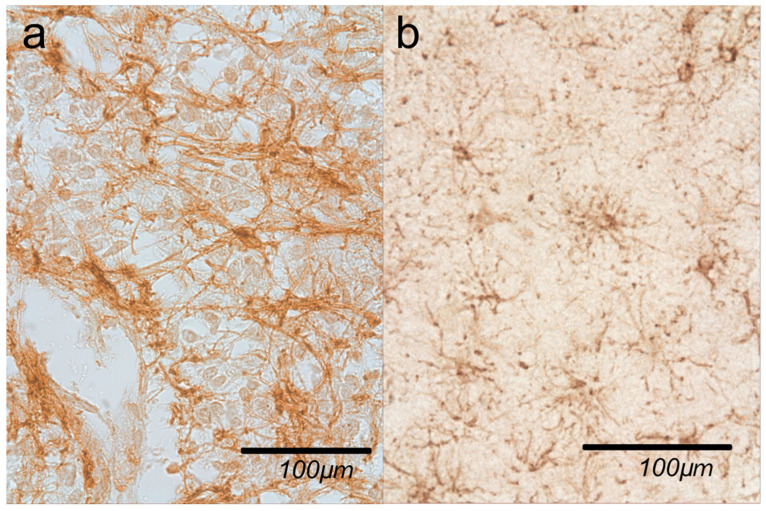
(**a**) Astrocytes in pineal gland parenchyma; (**b**) positive control for anti-GFAP antibody—typical astrocytes in brain tissue. Immunohistochemical staining with anti-GFAP antibody.

For one more positive control, we tested anti-GFAP antibodies on brain tissue; in Figure 7, we can see the typical astrocytes. For secondary antibody testing, we replaced primary antibodies with a PBS buffer.

#### 4.2.3. Analysis

We analyzed the density of the astrocyte network and the organization of the PG lobules in different age and experimental groups. The density of the astrocytic network and the degree of lobular organization were evaluated using a semi-quantitative visual approach, as no standardized scoring system for these parameters in the human pineal gland is currently available.

Astrocytic network density was classified into three categories: *light*, *medium*, and *dense*. *Light* was defined as sparse and discontinuous GFAP-positive structures, *medium* as a clearly detectable but moderate network, and *dense* as a compact and extensive GFAP-positive network.

Lobular organization was categorized as *intact*, *partially intact*, or *disrupted*, based on the degree of preservation of the typical lobular architecture. *Intact* referrs to well-defined and clearly delineated lobules, *partially intact* to altered, but still recognizable lobular structures, and *disrupted* to largely lost or indistinct lobular organization.

**Figure 8:**
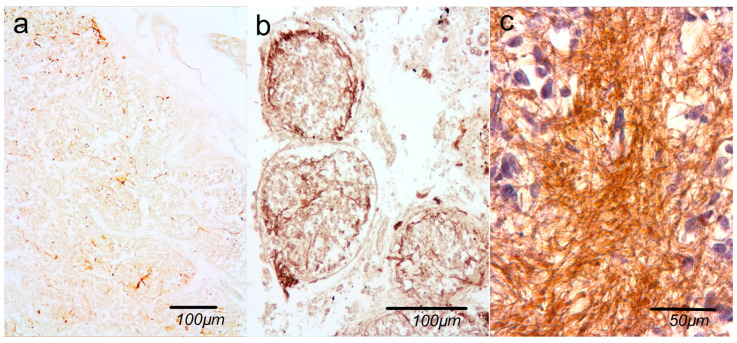
Astrocyte network organization: (**a**) *light* astrocyte network—sparse and discontinuous GFAP-positive structures; (**b**) *medium dense* astrocyte network—clearly detectable but moderate network; (**c**) *dense* astrocyte network—compact and extensive GFAP-positive network. Immunohistochemical staining with anti-GFAP antibody.

**Figure 9:**
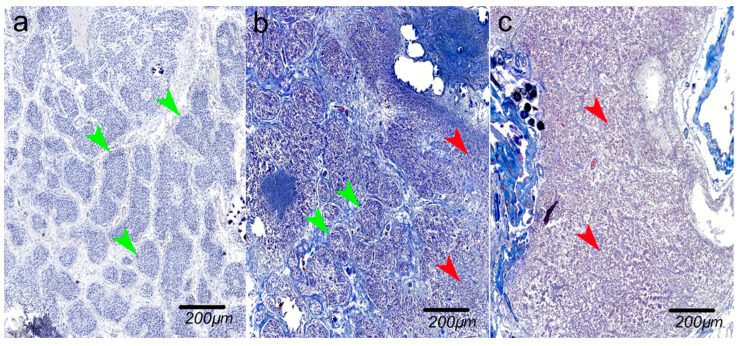
Lobular organization of parenchyma (**a**) *intact*—well-defined and clearly delineated lobules, gallocyanin staining; (**b**) *partially intact*—still recognizable lobular structures, Mallory staining; (**c**) *disrupted*—indistinct lobular organization, Mallory staining. Green arrows show intact lobules, red arrows show disrupted lobules.

All evaluations were performed consistently across samples under identical conditions. Examples of all variants are shown in Figure 8 and Figure 9.

#### 4.2.4. Statistical Analysis

Statistical analyses were performed in R/RStudio (version 4.5.3). Due to the small number of participants and nominal data, Fisher’s exact test was applied. Associations between health status and both lobular structure and GFAP distribution were examined. Using the same procedure, the association between lobular structure and GFAP distribution was also assessed. Results were considered statistically significant at *p* < 0.05. Effect magnitudes were calculated using Cramér’s V to quantify the strength of associations and were interpreted according to J. Cohen (2013) as small (0.1), medium (0.3), and large (0.5) [48].

## 5. Conclusions

By analyzing the lobular structure and astrocytic network of the human pineal gland (PG), we have identified two apparently distinct pathways of normal aging. In the first one, an increase in the number of astrocytes within the pineal parenchyma is observed, suggesting a partial compensatory role for astrocytes in maintaining pinealocyte function. In the second pathway, disruption of the lobular architecture appears to result in astrocytic atrophy and a decline in the functional integrity of all pineal components. These observations may explain our findings that the combination of a *disrupted* lobular structure and a *light* astrocytic network is the most common pattern, whereas the *dense* astrocytic network variant is found exclusively in structurally intact lobules of older individuals. Notably, the lobular organization of the pineal gland itself is highly variable and, apart from a slight tendency towards structural disruption with age, does not show a strong age-related pattern.

Another indicator of pineal degeneration is the presence of glial cysts, which are commonly observed in the pineal gland across the examined age range. Although typically asymptomatic, these cysts can significantly reduce the volume of the functional parenchyma. Notably, they are most commonly associated with the above-mentioned combination of a *disrupted* lobular structure and a *light* astrocytic network.

Based on our findings, we propose that pathological conditions may contribute to structural degeneration of the pineal gland and a subsequent decline in melatonin production; however, normal aging appears to be the primary driver of this involutional process.

## Figures and Tables

**Table 1:** Pathological variability—lobular structure organization and organization of the astrocyte network.

	State	Group				
		*Control*	*SCH*	*ALK*	*AD*	*VD*
Lobular Structure	*intact*	9 (25%)	3 (27.3%)	-	-	-
	*partly intact*	9 (25%)	1 (9.1%)	4 (57.1%)	3 (30%)	-
	*disrupted*	18 (50%)	7 (63.6%)	3 (42.9%)	7 (70%)	5 (100%)
GFAP-distribution	*light*	18 (50%)	7 (63.6%)	5 (71.4%)	8 (80%)	5 (100%)
	*medium dense*	15 (41.7%)	3 (27.3%)	-	1 (10%)	-
	*dense*	3 (8.3%)	1 (9.1%)	2 (28.6%)	1 (10%)	-

**Table 2:** Coincidence analysis lobular structure—GFAP distribution.

Lobular Structure—GFAP Distribution	*Control*	*SCH*	*ALK*	*AD*	*VD*
*intact—light*	1 (2.8%)	1 (9.1%)	-	-	-
*intact—medium dense*	5 (13.9%)	1 (9.1%)	-	-	-
*intact—dense*	3 (8.3%)	1 (9.1%)	-	-	-
*partly intact—light*	3 (8.3%)	1 (9.1%)	2 (28.6%)	3 (30%)	-
*partly intact—medium dense*	6 (16.7%)	-	-	-	-
*partly intact—dense*	-	-	2 (28.6%)	-	-
*disrupted—light*	14 (38.9%)	5 (45.4%)	3 (42.8%)	5 (50%)	5 (100%)
*disrupted—medium dense*	4 (11.1%)	2 (18.2%)	-	1 (10%)	-
*disrupted–dense*	-	-	-	1 (10%)	-

**Table 3:** Glial cysts findings in pineal gland.

Group	Number of Patients	Number of Glial Cysts	Average Age in the Group
Control	36	7	68.5
SCH	11	0	57.5
ALK	7	1	52.6
ALZ	10	1	78.4
VD	5	2	73.8

## Data Availability

The original contributions presented in this study are included in the article. Further inquiries can be directed to the corresponding author.

## Abbreviations

The following abbreviations are used in this manuscript:

PG	Pineal gland
GFAP	Glial fibrillary acidic protein
ALK	Alcoholism
SCH	Schizophrenia
ALZ	Alzheimer disease
VD	Vascular dementia
CNS	Central nervous system
BBB	Blood–brain barrier
